# A mathematical model of coronary blood flow control: simulation of patient-specific three-dimensional hemodynamics during exercise

**DOI:** 10.1152/ajpheart.00517.2015

**Published:** 2016-03-04

**Authors:** Christopher J. Arthurs, Kevin D. Lau, Kaleab N. Asrress, Simon R. Redwood, C. Alberto Figueroa

**Affiliations:** ^1^Division of Imaging Sciences and Biomedical Engineering, King's College London, King's Health Partners, St. Thomas' Hospital, London, United Kingdom;; ^2^King's College London British Heart Foundation Centre of Excellence, The Rayne Institute, St. Thomas' Hospital Campus, London, United Kingdom; and; ^3^Departments of Surgery and Biomedical Engineering, University of Michigan, Ann Arbor, Michigan

**Keywords:** coronary flow, exercise, mathematical model, metabolic control, autonomic control

## Abstract

This work presents a mathematical model of the metabolic feedback and adrenergic feedforward control of coronary blood flow that occur during variations in the cardiac workload. It is based on the physiological observations that coronary blood flow closely follows myocardial oxygen demand, that myocardial oxygen debts are repaid, and that control oscillations occur when the system is perturbed and so are phenomenological in nature. Using clinical data, we demonstrate that the model can provide patient-specific estimates of coronary blood flow changes between rest and exercise, requiring only the patient's heart rate and peak aortic pressure as input. The model can be used in zero-dimensional lumped parameter network studies or as a boundary condition for three-dimensional multidomain Navier-Stokes blood flow simulations. For the first time, this model provides feedback control of the coronary vascular resistance, which can be used to enhance the physiological accuracy of any hemodynamic simulation, which includes both a heart model and coronary arteries. This has particular relevance to patient-specific simulation for which heart rate and aortic pressure recordings are available. In addition to providing a simulation tool, under our assumptions, the derivation of our model shows that β-feedforward control of the coronary microvascular resistance is a mathematical necessity and that the metabolic feedback control must be dependent on two error signals: the historical myocardial oxygen debt, and the instantaneous myocardial oxygen deficit.

## Abstract (precis)

This paper presents a new mathematical model of the dynamic control of coronary resistance. It shows a remarkable ability to predict coronary flow in an exercising patient, which would otherwise be impossible, and provides new insight into the purpose and action of the coronary flow control systems. It is applicable as part of a controlled boundary condition at the coronary outlets of a three-dimensional Navier-Stokes simulation of hemodynamics.

## NEW & NOTEWORTHY

*This paper presents a new mathematical model of the dynamic control of coronary resistance. It shows a remarkable ability to predict coronary flow in an exercising patient, which would otherwise be impossible, and provides new insight into the purpose and action of the coronary flow control systems. It is applicable as part of a controlled boundary condition at the coronary outlets of a three-dimensional Navier-Stokes simulation of hemodynamics*.

the mass of oxygen delivered to the left-ventricular myocardium (O_2_D) is defined to be the product of coronary blood flow and arterial blood oxygen content (34). It is physiologically maintained at a value very close to that of the left-ventricular myocardial oxygen demand (MV̇o_2_) (6, 65). Such close matching requires a delicate balancing of coronary intrinsic and extrinsic control systems, which operate together to achieve rapid and accurate adjustment of O_2_D in response to changes in MV̇o_2_ (42).

At constant mean perfusion pressure, coronary flow is adjusted by control systems that modify coronary resistance. Such control systems fall into two categories: those that are feedforward control mechanisms, and those that operate via feedback. The former are anticipative mechanisms, which, based on neural signaling at the onset of exercise, trigger coronary vasodilation to preempt a portion of the expected myocardial oxygen supply-demand discrepancy before it arises (65, 25, 21, 62, 42). Feedforward control of the proximal and microvascular coronary resistances and, possibly to some extent, coronary vascular compliance, *C*_im_, is via neural impulse (21, 24). Sympathetic innervation of the coronary vessels affects vascular tone via adrenergic signaling. α- And β-adrenoceptors in the vasculature are responsible for vasoconstriction and vasodilation, respectively. β-Vasodilation appears to be responsible for ∼25% of the hyperemia observed during exercise (65). The second category of control, feedback, is characterized by the monitoring and countering of some error signal to fine-tune the response (21). For the myocardial blood supply, the feedback hypothesis (21, 64, 2) derives from the fact that coronary vessels vasodilate in correlation with increases in myocardial oxygen consumption independently of neural signaling, but the exact mechanisms are poorly understood (64, 56). While several previous hypotheses have been rejected as inadequate, including responses to adenosine or nitric oxide, and the involvement of K^+^ ATP channels (65), a current and promising *hypothesis 1* is that red blood cells themselves act as local O_2_ tension sensors, affecting vasodilation by releasing ATP in response to reduced O_2_ tension (24).

The physiological mechanisms which underlie these control systems are incompletely understood, but it is clear that they are complex, highly integrative, and nonlinear, which complicates their study. Mathematical models in which we can probe and control arbitrary system components and variables are an established and powerful way of studying complexity, enabling investigations that would be otherwise difficult or impossible and potentially allow predictions to be made. Additionally, there is interest in computational study of the three-dimensional hemodynamics in the coronary arteries, particularly in the presence of epicardial coronary artery disease, both for the purposes of basic research and for applications closer to the clinic (61). The lack of appropriate coronary boundary condition models for computational fluid dynamics (CFD) simulations, which are capable of responding in a physiological manner to changes in the cardiovascular environment, limits the fidelity of the results during physiological transitions such as that between rest and exercise, or of body orientation.

In this work, we introduce a zero-dimensional lumped parameter network (LPN) mathematical coronary flow control model (CFCM) representing the integrated behavior of these mechanisms, at an appropriate level of abstraction that can be used to simulate the coarse-scale operation of the control systems in terms of their ultimate impact on the delivery of oxygen to the myocardium. In addition to the coronary arteries, the simulated environment includes a model of the left ventricle and the aorta, from which cardiac workload, and thus approximate myocardial oxygen demand, are computed and passed to the CFCM. In this environment we will assess the ability of the proposed CFCM to reproduce several physiologically observed phenomena, including realistic beat-to-beat coronary flow patterns at rest and at hyperemia and coronary flow as recorded in human subjects during a transition from rest to exercise. We validate the model using three-dimensional CFD simulation, which allows computation of coronary flow velocity, which can be directly compared with patient Doppler flow velocity data.

### Previous Modeling Investigations

#### Coronary flow control models.

There are several previous models of coronary control. Miyashiro and Feigl (42) determined coronary flow from by an imposed MV̇o_2_ and coronary venous partial pressures of O_2_ and CO_2_, concluding that the speed and accuracy of the coronary flow response to MV̇o_2_ perturbations are improved upon by the addition of stronger β-feedforward or the weakening of α-feedback control, that a greater feedback gain increases the speed of the flow response but can lead to instability, and that including a time delay in the α-feedforward system improves stability. However, this model has no concept of vascular resistance, so flow was directly imposed upon the system, and it cannot respond to historical oxygen supply deficits such as occurs in reactive hyperemia.

Dankelman et al. (12) created a mathematical model of coronary resistance changes in response to changing myocardial oxygen partial pressure, based on experimental data quantifying the correlation between MV̇o_2_ and coronary flow at any given perfusion pressure (67). In a series of investigations, they studied dynamic responses to perturbation in the coronary circulation of the goat, discovering that their model predicted observations that the coronary resistance took longer to complete its response to changes in MV̇o_2_ at high constant perfusion pressure than at low, and at constant flow, the response took longer still (12). In subsequent work in goats, they observed that at constant MV̇o_2_ step changes in constant pressure or flow elicited a change in the resistance that took longer to complete when the step was an increase as opposed to a decrease. Their model was not able to reproduce this effect without using a differently parameterized models for each direction of step. The model was later combined with mechanical models to examine the fast and slow phases of the coronary resistance response to perturbation, identifying that intramyocardial compliance with varying venous resistance or the vascular waterfall model (18) with a small compliance could both explain the fast phase (14). Similar perturbation responses were later examined in dogs, where the responses were seen to be around four times faster and in which the directional sensitivity of responses to perfusion pressure or flow steps was absent (15).

Arciero et al. (2) performed a mechanistic study of metabolic, myogenic, and shear stress control systems using a zero-dimensional model of a generic vascular bed, with compartments representing the capillaries, large and small arterioles, and venules. They investigated the hypothesis that red blood cells act as oxygen saturation sensors, releasing ATP as a signaling molecule when low O_2_ saturation is detected. The ATP signaling information is then conducted upstream of the site at which the red blood cells detected low saturation, assuming an exponential decay of the signal with distance, causing vasodilation. This model adjusts the resistances of vessels in each compartment by adjusting their nominal diameters in response to the combination of the shear, myogenic, and conducted metabolic responses. At low levels of oxygen consumption, the model agrees with the relationship between O_2_ supply and demand observed in dogs. The relative importance of the shear, metabolic, and myogenic mechanisms observed in this model supports the hypothesis that metabolic control is by far the most important of the mechanisms in terms of being the overall determinant of blood flow. This model does not include feedforward control, and, as the authors note, in the form presented it is not suitable for use at high levels of oxygen demand.

The only existing work that we are aware of in the literature that involves a model for coronary flow control applied as a boundary condition for Navier-Stokes multidomain hemodynamics was produced by Kim et al. (32), whose model makes adjustments to coronary flow in response to changes in myocardial oxygen demand. However, the model of Kim et al. model must be seen as being more akin to feedforward control than to feedback; system perturbations in myocardial oxygen demand are converted directly, and apparently instantaneously, into changes in coronary resistance, which are guaranteed to match oxygen supply with oxygen demand. In the present work, we introduce a true feedback model, while also including feedforward components.

#### Structural models of the coronary arteries and of myocardial perfusion.

While there are a limited number of previous modeling investigations into coronary control systems, many workers have created structural models of the coronary system. Mynard and Nithiarasu (46) modeled a gradient of extravascular compression along a one-dimensional coronary vessel, representing the differing compression along its length. Later, Mynard et al. used LPN models that consider the differing extravascular compression within different layers of the myocardium (45), and resistances dependent on regional blood occupancy (47).

Cookson et al. (9) studied perfusion of the myocardium as a homogenized poroelastic medium, in which the permeability tensors were derived from the structure of the intramyocardial coronary tree. This permitted investigation of the impact of structural properties of the myocardium upon perfusion, neglecting contraction, although inclusion of contractile deformation is also possible (55, 54). For a general review of this topic, see Nolte et al. (49), and for a review of approaches to computational coronary flow modeling, excepting control, see Lee and Smith (36).

## METHODS

### Overview

**Fig. 1.**
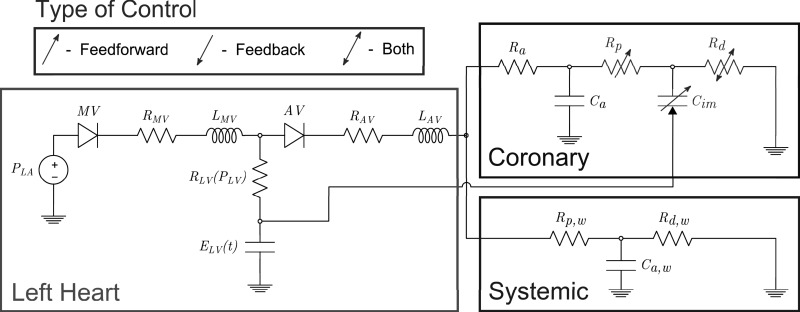
The zero-dimensional simulation circuit, divided into left heart, coronary, and systemic circulation components. The time-varying left-ventricular elastance, *E*_LV_ (*t*), is responsible for left-ventricular pressure generation. Left atrial pressure, *P*_LA_, is maintained constant in this model. The mitral valve, MV, and aortic valve, AV, are each represented by a diode, resistor, R_*V_, and inductor, L_*V_. The left ventricular internal resistance *R*_LV_ (*P*_LV_) is nonlinear, dependent on the current LV pressure. In the coronary circulation, extravascular compression of the intramyocardial vasculature is modeled by passing left ventricular pressure to one node of capacitor *C*_im_. This capacitor, together with the proximal and microvascular resistances *R*_p_ and *R*_d_, experience adjustment due to the control systems. See *Lumped Models of the Heart and Vascular Beds* for further details.

We present and evaluate our CFCM model using both a zero-dimensional LPN model, shown in Fig. 1, and a zero-dimensional-three-dimensional coupled multidomain model (69), in which the coronary, left-heart, and systemic components of Fig. 1 are attached to, respectively, coronary, aortic-inlet, and the systemic boundaries of a three-dimensional model, with the coronary circuit providing a controlled boundary condition. The deformability of the three-dimensional vessel walls is simulated using the coupled momentum method (22). The software used for each are independent of one another, with the former being a custom MATLAB program, and the latter being CRIMSON (CardiovasculaR Integrated Modeling and SimulatiON) (1), our highly parallel, stabilized (SUPG), incompressible Navier-Stokes multidomain finite element software, written using Fortran, C++, and MPI, implementing methods described previously (69, 68, 73); we actively develop CRIMSON in our research group, and it is suitable for use on computer hardware ranging from laptops to national-scale supercomputers. The software models blood as an incompressible, viscous, Newtonian fluid. This is appropriate, as in vessels that are large compared with the diameter of red blood cells, blood is Newtonian (48). The MATLAB program generates many thousands of cardiac cycles in a short period of time. The multidomain model is far more computationally demanding, but the realism of the simulation is greater, in terms of the inclusion of blood momentum, arterial compliance, and pressure-wave propagation, but most importantly, it has the capacity for including multiple, three-dimensional, patient-specific coronary vessels, each terminated with an independent instance of our controlled coronary LPN for its myocardial perfusion territory and interacting with one another naturally via the fluid physics of the three-dimensional domain; see Fig. 8 for our idealized coronary test geometry. The use of two independent implementations also provides a degree of verification of the results.

### Lumped Models of the Heart and Vascular Beds

Simulation of realistic hemodynamics requires a method of generating pulsatile aortic inflow. To achieve this, we make use of an existing lumped parameter model of the left side of the heart (35), shown in Fig. 1. This model relies on LV volume tracking, together with a cyclic time-varying elastance function *E*_LV_(*t*) (50), which drives the generation of pressure by adjusting the left ventricular elastance denoted by variable capacitor *E*_LV_ in Fig. 1. In addition to generating pulsatile inflow, a key consideration is that this model produces left-ventricular pressure-volume (PV) loops, which we can use to compute myocardial work and thus estimate MV̇o_2_, which is an input for the coronary flow control system; see *Computing the Myocardial Oxygen Consumption*.

In our zero-dimensional experiments, this heart model is directly coupled to a three-element Windkessel model (72), representing the systemic circulation, and the resulting aortic pressure is used to drive flow through the resistance-controlled coronary LPN shown in Fig. 1. In our multidomain studies, the LPN models are attached to the appropriate boundary surfaces of the vascular geometry, and the coupling between the heart and the LPNs occurs via the hemodynamics within the vessels. See Fig. 8 for a the three-dimensional model and its boundary surfaces.

The lumped parameter coronary model depicted in Fig. 1 is of a standard design (38, 31, 32, 30, 45), modified to include control of several of its components. These models were developed to reproduce the compression of the intramyocardial compliance vessels during systole, and the associated intramyocardial pumping effect that produces the characteristic coronary flow patterns (58).

### Modeling the Physiological Mechanisms of Coronary Flow Control

Before giving the mathematical formulation of the model, we briefly explain the requirements that we have derived from the current understanding of the physiology.

#### Feedforward mechanisms.

Feedforward α-vasoconstriction predominantly effects vessels of diameter >100 μm, whereas feedforward β-vasodilation occurs in vessels of diameter <100 μm (7, 21). Thus, with reference to the coronary model shown in Fig. 1, we propose that α-feedforward vasoconstriction control should affect the more proximal microvascular resistor, *R*_p_, and that β-feedforward vasodilation should affect the more distal resistor, *R*_d_. The occurrence of α-vasoconstriction at the onset of exercise has been described as “paradoxical” (21), but one postulated explanation is that it acts to improve overall coronary perfusion by reducing systolic retrograde coronary flow (43). Vascular compliance reduction, which has been proposed to be an important effect of α-feedforward vasoconstriction (65), could therefore affect the intramyocardial vessel compliance, *C*_im_, as there are vessels of diameter >100 μm within the myocardium (51). To the best of our knowledge, there is no dynamic control model for *C*_im_ in the literature; we will use manual adjustments of *C*_im_ to consider the possibility of it being a physiologically controlled parameter.

#### Feedback mechanisms.

To create the feedback component of our model, we require the concept of a myocardial oxygen supply error signal (OSES), a hypothetical signal that reflects any mismatch in the oxygen supply-demand ratio within the myocardium. We do not make any mechanistic claims regarding how it arises. We make use of the facts that due to the normally low oxygen content of coronary venous blood, the control systems adjust coronary flow so that it closely follows myocardial oxygen demand (4, 70), and that the site of metabolic control is those vessels of diameter <100 μm (28). Thus we assign it to our *R*_d_ resistance in the coronary circuit (Fig. 1).

Related feedback control systems in the coronary vasculature respond to local changes in vessel wall shear and circumferential stress. Endothelial cells detect elevated shear stress and signal to the vascular smooth muscle to trigger vasodilation. Changes in circumferential stress are detected and countered by the smooth myocytes themselves; this is known as the myogenic mechanism (10, 59, 71). Although these mechanisms are separate, their actions influence one another. Thus, under physiological conditions, the control mechanisms ultimately combine to scale coronary blood flow to the myocardial oxygen demand.

The proposed CFCM represents a lumping together of these feedback mechanisms: metabolic, myogenic, and shear control. This is not only a suitable phenomenological approach to the concerted action of the feedback mechanisms but also an expedient one, given the fact that we parameterize our CFCM using experimentally observed coronary flow responses to perturbation, which necessarily represent the net effect of these mechanisms. A similar argument was used to justify the lumping of feedback mechanisms in the related zero-dimensional modeling work of Miyashiro and Feigl (42).

#### Summary of control locations within the LPN.

The parameters dynamically adjusted by our CFCM are the resistances *R*_p_ and *R*_d_. *R*_p_ experiences feedforward vasoconstriction, so here we are implicitly assuming that *R*_p_ approximates the resistance of small vessels of diameter >100 μm. Similarly, *R*_d_ approximates the resistance in microvessels of diameter <100 μm (65) and experiences both β-feedforward vasodilation and metabolic feedback control. Note that because the model is lumped, the spatial ordering of the components should not be interpreted rigidly; for example, *C*_im_ is a lumped compliance of nearby vessels, including portions of those that contribute the resistances *R*_p_ and *R*_d_. Note that the majority of the compliance exists within the capillaries, so *C*_im_ and *R*_d_ have more “lumped overlap” than *C*_im_ and *R*_p_ (8, 57).

### Mathematical Model of Coronary Flow Control

We now explain the construction of our CFCM. We state our guiding principles assumptions as follows.

*1*) Myocardial oxygen supply should closely match the myocardial oxygen demand.

*2*) Control of coronary flow should primarily be via a feedback mechanism that evaluates, and acts to counter, any discrepancies in the oxygen demand.

*3*) The control system should take into account the historical state of the system, in a manner that allows it to repay any oxygen “debts” that have arisen.

*4*) We make the modeling assumption that all changes in the volume of oxygen extracted from the coronary blood by the myocardium are facilitated solely by changes in coronary flow; in particular, we assume that coronary venous blood oxygen content and myocardial O_2_ extraction per unit volume of blood delivered to the myocardium are constant.

These principles are reasonable, as coronary flow closely follows cardiac work (4, 56), because of the experimentally observed state of reactive hyperemia, which is known to occur for a period after a coronary occlusion is reversed (39), and because the change in coronary venous O_2_ partial pressure between rest and exercise is small, relative to the arteriovenous difference in O_2_ partial pressure (42). The final point means that varied oxygen delivery, as opposed to varied oxygen extraction per unit volume of blood, is the dominant physiological source of varied total oxygen extraction by the myocardium, and it is this that we model, but we note that in reality there is a well-documented degree of variation in extraction.

With *principle 4* in mind, we begin by writing an expression for the instantaneous myocardial oxygen deficit at time *t*(*s*), *h*(*t*) (cm^3^/s), in terms of the instantaneous myocardial oxygen demand, MV̇o_2_(*t*) (cm^3^/s), the volumetric coronary flow *Q*_cor_(*t*) (cm^3^/s) and the (coronary-extractible) blood oxygen content volume proportion, γ,

(1) $$h\left(t\right)=\text{M}\overset{˙}{\text{V}}{\text{O}}_{2}\left(t\right)-\gamma Q_{\text{cor}}\left(t\right).$$

This is similar to the statements that have guided previous modeling attempts that oxygen supply must match the demand (12, 19). Because of *principle 3*, we must work with the total myocardial OSES,

(2) $$H(t)\;:=\int_{0}^{t}h\left(\tau \right)d\tau ,$$

[units (cm^3^)], as opposed to the instantaneous value of *h*. We introduce the equation

(3) $$\frac{d^{2}H\left(t\right)}{dt^{2}}=-k_{fb}H\left(t\right)-g\frac{dH\left(t\right)}{dt},$$

which describes damped harmonic motion of the total myocardial OSES. The positive constants *k*_*fb*_ (s^−2^) and *g* (s^−1^) are the feedback gain and the damping coefficient, respectively. This equation represents the tendency for *H* to be returned to zero by the control systems, and allows variation in MV̇o_2_(*t*) to be taken into account, thus addressing *principles 1* and *2*. *Equations 1*, *2*, and *3* can be combined to give

(4) $$\frac{dQ_{\text{cor}}\left(t\right)}{dt}=k_{\text{fb}}\gamma^{-1}H\left(t\right)+g\gamma^{-1}\frac{dH\left(t\right)}{dt}+\gamma^{-1}\frac{d\text{M}\overset{˙}{\text{V}}{\text{O}}_{2}\left(t\right)}{dt}.$$

We now describe how this expression can be used to control coronary blood flow in a LPN coronary model, as shown in Fig. 1. The parameter that must be controlled is the total resistance *R*_cor_(*t*) = *R*_a_ + *R*_p_(*t*) + R_d_(*t*) (N·s·cm^−5^) of the circuit; this represents coronary vasoconstriction or vasodilation in the physiological system. Thus we need to relate the control described by *Eq. 4* to *R*_cor_(*t*). For notational simplicity, we define *S*_cor_(*t*) = [*R*_cor_(*t*)]^−1^, and with *P*_cor_(*t*) (N/cm^2^) the coronary perfusion pressure, we write

(5) $$S_{\text{cor}}\left(t\right)P_{\text{cor}}\left(t\right)=Q_{\text{cor}}\left(t\right).$$

We assume that the control system follows *Eq. 5* to convert a desired change in flow into the appropriate modification of coronary resistance. We further assume that this relationship operates with a fixed mean coronary perfusion pressure *P*_cor_(*t*): = *P̄*_cor_, as opposed to an instantaneous pressure. In this work, we fix *P̄*_cor_ = 100 (mmHg) ≈ 1:33322 (N/cm^2^); we might speculate that this value could be hard-wired into the physiological control system as appropriate for a young, healthy individual at rest, although it may vary in exertion, or with age or disease. Inserting this assumption, differentiating *Eq. 5* with respect to *t*, and combining with *Eq. 4*, we arrive at the equation for resistance control,

(6) $$\frac{dS\left(t\right)}{dt}{\overset{¯}{P}}_{\text{cor}}=k_{\text{fb}}\gamma^{-1}H(t)+g\gamma^{-1}\frac{dH\left(t\right)}{dt}+\gamma^{-1}\frac{d\text{M}\overset{˙}{\text{V}}{\text{O}}_{2}\left(t\right)}{dt}.$$

Note that *Eq. 6* indicates that control of coronary resistance is dependent on two feedback error signals: the total historical myocardial OSES *H*, and upon the instantaneous myocardial oxygen deficit *h* = $\frac{dH}{dt}$. Interestingly, we discover that it is also directly dependent on changes in MV̇o_2_, with $\gamma^{-1}\frac{d\text{M}\overset{˙}{\text{V}}{\text{O}}_{2}\left(t\right)}{dt}$ being a feedforward term which affects resistance as β-feedforward should, a detail that we will return to in *Adrenergic feedforward*. The resistance change dictated by this equation is assigned to the coronary microvasculature, viz. *R*_d_(*t*); here, a minimum value can be set, for example to represent microvascular dysfunction. It should not be allowed to less than the coronary venous resistance (noting that the coronary venous resistance must also be considered to be amalgamated with *R*_d_). This derivation completes the model of metabolic feedback and β-adrenergic feedforward control of coronary microvascular resistance.

The α-adrenergic feedforward control is modeled solely in terms of its effect on the proximal microvascular resistance, *R*_p_(*t*). Writing *S*_p_(*t*) = [*R*_p_(*t*)]^−1^, we control *R*_p_(*t*) via the equation

(7) $$\frac{dS_{\text{p}}\left(t\right)}{dt}{\overset{¯}{P}}_{\text{cor}}=\gamma^{-1}k_{\text{f f}}^{\text{p}}\frac{d\text{M}\overset{˙}{\text{V}}{\text{O}}_{2}\left(t\right)}{dt}.$$

where *k*_ff_^p^ < 0 is the (dimensionless) gain of the α-feedforward control system.

### Computing the Myocardial Oxygen Consumption

To apply *Eq. 6*, a model for the myocardial oxygen demand MV̇o_2_(*t*) is needed. The amount of oxygen required by the myocardium should be related to the cardiac work. This can be computed from the area enclosed by the ventricular PV loop, determined from the heart model shown in Fig. 1. We limit our consideration of myocardial work, and thus of MV̇o_2_(*t*), to the left ventricle, although our approach could be equally applied to the right ventricle.

Suppose that we index the cardiac cycles as {*c*_*i*_}_*i*=1,2,3,…_, with cycle *c*_*i*_ commencing at the time of onset of the *i*th contraction of the myocardium, *t*_*i*_ seconds. Measuring the pressure in Pascals and the volume in cubic meters, we denote the PV area for cycle *c*_*i*_ by PVA(*c*_*i*_) (Joules), and we assume that metabolism of 1 ml of O_2_ provides 20 J of energy (29, 11). Now we let $BO_{2}(c_{i})=\int_{t_{i}}^{t_{i+1}}M\overset{˙}{V}O_{2}(\tau )dt$ be the total O_2_ requirements of the left ventricle during cycle *c*_*i*_. The relationship between PVA(*c*_*i*_) and BO_2_(*c*_*i*_) for the LV can be approximated as PVA(*c*_*i*_) = BO_2_(*c*_*i*_) from experimental data (29). From this, we can compute BO_2_(*c*_*i*_) and convert it into a continuous, piecewise-linear form, which for *t*_*i*_ ≤ *t* < *t*_*i*_ + 1, is given by

(8) $$\text{M}\overset{˙}{\text{V}}{\text{O}}_{2}\left(t\right)=\frac{{\text{BO}}_{2}\left(c_{i-2}\right)}{t_{i-1}-t_{i-2}}\frac{t_{i+1}-t}{t_{i+1}-t_{i}}+\frac{{\text{BO}}_{2}\left(c_{i-1}\right)}{t_{i}-t_{i-1}}\frac{t-t_{i}}{t_{i+1}-t_{i}}.$$

Note that the use of *Eq. 8* implies that the value of MV̇o_2_(*t*), which we use in our control system is based on the oxygen consumption during the previous two cardiac cycles.

### Model Parameters

#### Control system parameters.

When designing a mathematical model of such a complex system, one must consider the quantity and quality of experimental data available for parameterization of the system. It is for this reason that we have attempted to construct a model with the fewest parameters possible, while still being capable of reproducing physiological phenomena. *Equation 6* contains four parameters explicitly and another three implicitly. The explicit parameters are the feedback gain *k*_fb_, the damping coefficient *g*, the mean pressure *P̄*_cor_, and the coronary-extractible arterial blood oxygen content γ. The implicit parameters are the myocardial efficiency and the energy associated with the metabolism of 1 ml of O_2_, which are used in the computation of MV̇o_2_(*t*), and were discussed in *Computing the Myocardial Oxygen Consumption*. The final implicit parameter is the repayment cost of a historical oxygen debt, which is contained in *H*(*t*) and which we take to be equal to one. A future possibility here is to use an integration kernel in *Eq. 2* to modify the cost of repaying longer-standing oxygen debt to reproduce the physiological repayment ratio effect (39). This will be discussed further in *Reactive hyperemia*.

Of the explicit 1 parameters, γ = 0.125 is computed from coronary flow, MV̇o_2_, and coronary sinus blood oxygen content data; we take it to be the approximate gradient of MV̇o_2_ vs. coronary blood flow relationship shown in Fig. 10-5 of Ref. 4. We emphasize that is not the total blood oxygen content but rather that oxygen content that will be extracted as the blood passes through the myocardial vasculature. Opting to hold this constant is a simplification, but a reasonable one, as coronary blood oxygen extraction is constantly close to maximum (6, 70). *P̄*_cor_ was discussed in *Mathematical Model of Coronary Flow Control*; *k*_fb_ and *g* remain to be discussed. These final two parameters are tuned so that when the coronary perfusion pressure is sharply perturbed, the flow response is oscillatory with period 10–15 s and stabilizes in ∼30 s. This is based on experimental data in anesthetized dogs (16). While that experiment demonstrates an autoregulatory response, it should be noted to that the metabolic control, which we model here, is involved in this response (20) and that it is difficult to separate metabolic control from other autoregulatory mechanisms that are also involved, such as the myogenic vessel circumferential stress and the endothelial wall shear stress control systems (70).^1^ This parameterization will be performed in *Physiological Response to Perfusion Pressure Perturbation: a Parametrization Study*.

For the α-adrenergic feedforward control, modeled by *Eq. 7*, we take *k*_ff_^p^ = −0.1. In practice, this results in minor resistance increases with increases in MV̇o_2_.

#### LPN parameters.

LPN parameters are determined independently of the control model. The parameters for the heart model subunit shown in Fig. 1 are given in Table 1; their values are determined so as to produce physiological cardiac behavior (35). The basic (nonexercising) elastance function *E*_LV_ was parameterized by Lau and Figuera (35). These values are used for all simulations. The parameters for the systemic Windkessel model shown in Fig. 1 are specified in Table 2. These are tuned using the standard method of setting the total resistance according to a target mean aortic pressure, *C*_a,w_ is adjusted so that the pulse pressure is physiological, and the ratio of *R*_p,w_ to *R*_d,w_ is then determined such that diastolic pressure decay is physiological. The coronary parameters are determined so that the mean flow at rest is appropriate and so that the characteristic coronary systolic-diastolic flow patterns are achieved. These must be determined for each coronary; the values selected for our three-dimensional simulation (including the associated Windkessel models) were presented by Kim et al. (33), with the exception that we used a compliance of 5.4 × 102 cm^5^/N at the outlet at the descending aorta, as we found that this improved the pulse pressure. The values set and control ranges observed are shown in Tables 3 and 4.

**Table 1.** Component parameters for the heart model

	*P*_LA_	*R*_MV_	*R*_AV_	*R*_LV_ (*P*_LV_)	*L*_MV_	*L*_AV_
Value/units	0.05333 N/cm^2^	3.9 × 10^−5^ N·s·cm^−5^	1.0 × 10^−6^ N·s·cm^−5^	3.8 × 10^−7^ · *P*_LV_ N·s·cm^−5^	1.0 × 10^−6^ N·s·cm^−5^	1.0 × 10^−6^ N·s·cm^−5^

*P*_LV_ is the pressure within the left ventricle; the given value is equal to 4.0 mmHg. See text and Fig. 1 for the parameter associations with the circuit.

**Table 2.** Component parameters for the Windkessel model in the pure-LPN simulations

	*C*_a,w_	*R*_p,w_	*R*_d,w_
Value/units	1.3 × 10^2^ cm^5^/N	5.8 × 10^−4^ N·s·cm^−5^	2.0 × 10^−2^ N·s·cm^−5^

LPN, lumped parameter network. See text and Fig. 1 for the parameter associations with the circuit.

**Table 3.** Component parameters for the coronary model in the pure-LPN simulations

Parameter	*C*_a_	*C*_im_	*R*_a_	*R*_p_	*R*_d_
Value	4.5 × 10^−2^ cm^5^/N	2.7 × 10^−1^ cm^5^/N	3.2 × 10^−1^ N·s·cm^−5^	[6.4 × 10^−1^, 6.7 × 10^−1^] N·s·cm^−5^	[1.1, 1.8 × 10^1^] N·s·cm^−5^

Those that varied due to control are given as the ranges we observed. See text and Fig. 1 for the parameter associations with the circuit.

**Table 4.** Component parameters for the coronary models in the 3-dimensional simulations

	*C*_a_	*C*_im_	*R*_a_	*R*_p_	*R*_d_
*Coronary A*	3.4 × 10^−2^ cm^5^/N	2.9 × 10^−1^ cm^5^/N	2.0 N·s·cm^−5^	[6.4 × 10^−1^, 6.7 × 10^−1^] N·s·cm^−5^	[1.4, 6.9] N·s·cm^−5^
*Coronary B*	4.8 × 10^−2^ cm^5^/N	4.0 × 10^−1^ cm^5^/N	2.3 N·s·cm^−5^	[6.4 × 10^−1^, 6.7 × 10^−1^] N·s·cm^−5^	[0.7, 7.3] N·s·cm^−5^
*Coronary C*	4.8 × 10^−2^ cm^5^/N	4.2 × 10^−1^ cm^5^/N	2.2 N·s·cm^−5^	[6.4 × 10^−1^, 6.7 × 10^−1^] N·s·cm^−5^	[0.72, 7.7] N·s·cm^−5^

Those that varied due to control are given as ranges. See Fig. 8 for the referenced coronary boundaries.

### Human Data for use in Model Testing

A male patient with symptoms of exertional angina and documented coronary artery disease (>70% stenosis in a major epicardial vessel; in the case of this individual the stenosis severity was <80%) was recruited from a routine patient waiting list for percutaneous coronary intervention at St Thomas' Hospital. The patient was catheterized via the right radial artery using a 6-F arterial sheath to allow for supine bicycle exercise as previously described (37). Coronary blood flow velocity was acquired using a 0.014-ft. intracoronary wire (CombowirerXT; Volcano, San Diego, CA), positioned distal to the coronary stenosis, and aortic pressure was acquired using a pressure sensor 0.014-ft. Primewire Prestiger (Volcano), positioned in the aortic root. Informed consent was obtained, and the acquisition was approved by the institutional research ethics committee (08/H0802/39).

The recorded patient HR and aortic pressure data will be imposed as inputs to our model. The reason for doing this is that the two key determinants of myocardial work are the HR and cardiac afterload, so for our purposes, their imposition should be sufficient input to evaluate whether the control system can reproduce the associated coronary flow data. HR is imposed by adjusting the period of the elastance function, whereas peak systolic pressure is affected by adjusting *E*_max_, the maximum value of *E*_LV_. These two parameters are interlinked; at lower heart rates, a higher *E*_max_ will be required to achieve a given peak systolic pressure. For this reason, we empirically constructed the mathematical surface which maps HR and target peak systolic pressure to the required *E*_max_ that will, after stabilizing, give that target peak systolic pressure. This map must be reconstructed for each specific application.

## RESULTS

In this section, we present the controlled coronary flow dynamics in a number of applications designed to test different physiological aspects of our CFCM.

Our zero-dimensional results were computed using a timestep of 5 ms, and our multidomain simulations used a timestep of 1 ms, 93,425 mesh nodes, and a tolerance of 0.001 for convergence of the nonlinear iteration.

### Physiological Response to Perfusion Pressure Perturbation: a Parametrization Study

**Fig. 2.**
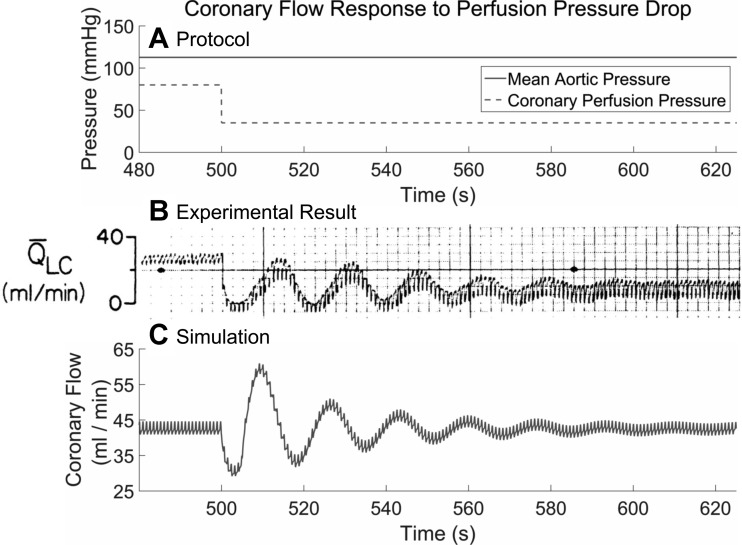
Comparison between the experimental and simulated response of the control system to a severe coronary perfusion pressure drop, without changes in the aortic pressure, as shown in *A*. The experimental result shown in *B* is for canine coronary flow and is reproduced with permission from the work of Canty and Klocke (5), who time averaged this flow using a built-in filter in their equipment. The oscillatory period and decay are in good agreement with the simulated coronary flow control model (CFCM) response, shown in *C*, to the same experimental protocol. The data in *C* were time averaged using a 5-s-window moving average filter. Note that the CFCM returns the flow to the baseline after the perturbation, but the experimental results show that such a severe drop in coronary perfusion pressure results in a reduced final flow. See *Physiological Response to Perfusion Pressure Perturbation: a Parametrization Study* for further details.

Canty and Klocke (5) demonstrated damped oscillations in coronary blood flow after a severe drop in coronary perfusion pressure from 80 to 35 mmHg (1.0665 to 0.4666 N/cm) in a cannulated canine left circumflex artery, while maintaining aortic pressure to avoid changing cardiac workload and thus maintaining MV̇o_2_; see Fig. 2. As the experimental work does not distinguish between the coronary control systems, it is appropriate to expect that our CFCM, which is phenomenological in nature, should be parameterized to give similar dynamics to the experimental case. To reproduce the experimentally observed oscillatory period and decay shown in Fig. 2, we found empirically that a feedback gain of *k*_fb_ = 91/225, and a damping coefficient *g* = 21/80, were appropriate.

Agreement between the experimental and simulation data is good; it is this result in particular that gives us confidence in the ability of our CFCM to produce physiological dynamics. However, note that the simulated flow returns to its preperturbation value, whereas the experimental flow does not. It is a well-known, incompletely explained phenomenon that coronary vasodilator reserve can remain during myocardial ischemia (20), with proposed explanations including reduced washout of vasoconstrictor metabolites or reduced regional myocardial contraction (5). We note that similar oscillations are observed over different pressure-perturbation ranges by other experimentalists (16, 44, 17).

### Model Behavior under Synthetic Conditions

#### Reproducing coronary flow profiles at rest and under stress.

The coronary arteries are unique in that they experience greater flow during diastole than during systole. The coronary LPN (38, 30, 33) that we base our work on is capable of reproducing this, but only when it is correctly parameterized. Since the present work involves dynamic adjustment of these parameters, it is important to demonstrate that this systolic-diastolic flow pattern is preserved by control adjustments in response to strong perturbations of MV̇o_2_.

**Fig. 3.**
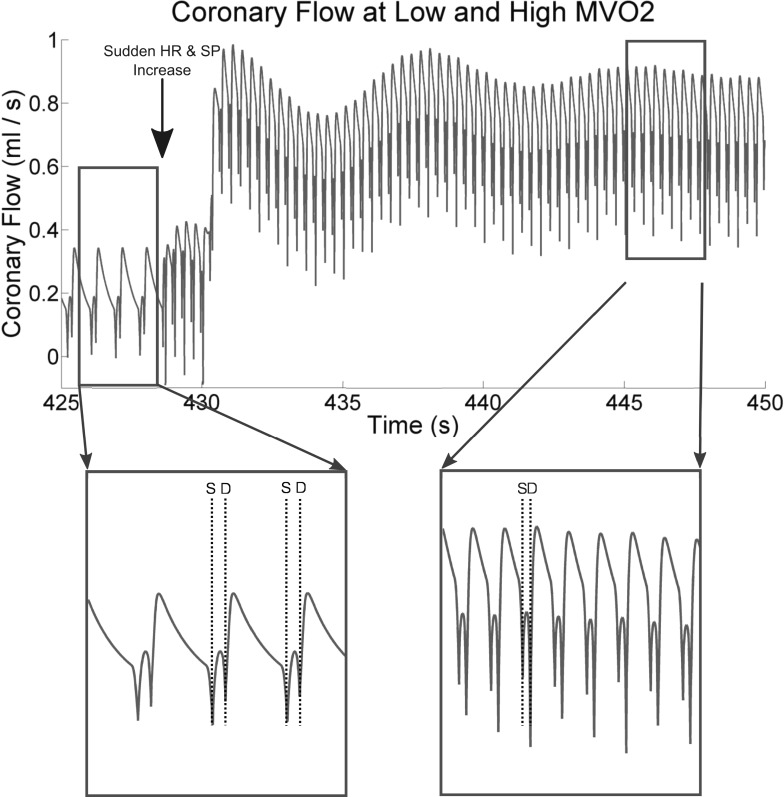
A demonstration that the systolic-diastolic flow patterns remain physiological, both at rest and under an approximation of exercise-stress, despite dynamic control of coronary lumped parameter network (LPN) resistances. A strong increase in heart rate (HR) and *E*_max_ is implemented at the point marked by a large vertical arrow on at *top*, causing the systolic pressure (SP), and thus MV̇o_2_, to rise. The control system responds by reducing the coronary resistance, so flow increases. Note that the low systolic (from “S” markers) and high diastolic (from the “D” markers) flows agree qualitatively with the human systolic-diastolic coronary flow pattern (for example, see Fig. 4 of Ref. 58). See the text of *Reproducing coronary flow profiles at rest and under stress* for more details.

To this end, Fig. 3 shows the results of a zero-dimensional experiment, using the circuit shown in Fig. 1, beginning at an imposed HR of 70 beats/min and a peak systolic pressure of 127 mmHg (1.6931 N/cm^2^) and being instantaneously switched to 180 beats/min with an *E*_max_, which eventually results in a stable systolic pressure of 200 mmHg (2.6664 N/cm^2^). This perturbation is quite extreme, and it does not perfectly represent exercise, because we do not attempt to make exercise-appropriate adjustments of the systemic arterial Windkessel model (see Fig. 1); this will affect cardiac afterload and thus MV̇o_2_. However, it demonstrates that, under modifications to the coronary resistances by the control system, the systolic-diastolic coronary flow pattern is maintained, and qualitatively agrees with the human flow velocity data at rest and at hyperemia (23).

We have found that the damping coefficient needs to be scaled according to the compliance of the intramyocardial vessels. The appropriate value of this parameter scales approximately inversely with the value of *C*_im_, but that the relationship is nonlinear and so should be tuned manually to give the oscillatory periods and decays of Fig. 2, once *C*_im_ has been set. Note that this observation suggests that the effective level of damping in the system can be increased by either increasing *g* or by decreasing *C*_im_.

#### The control response to a ramp of increasing cardiac workload.

**Fig. 4.**
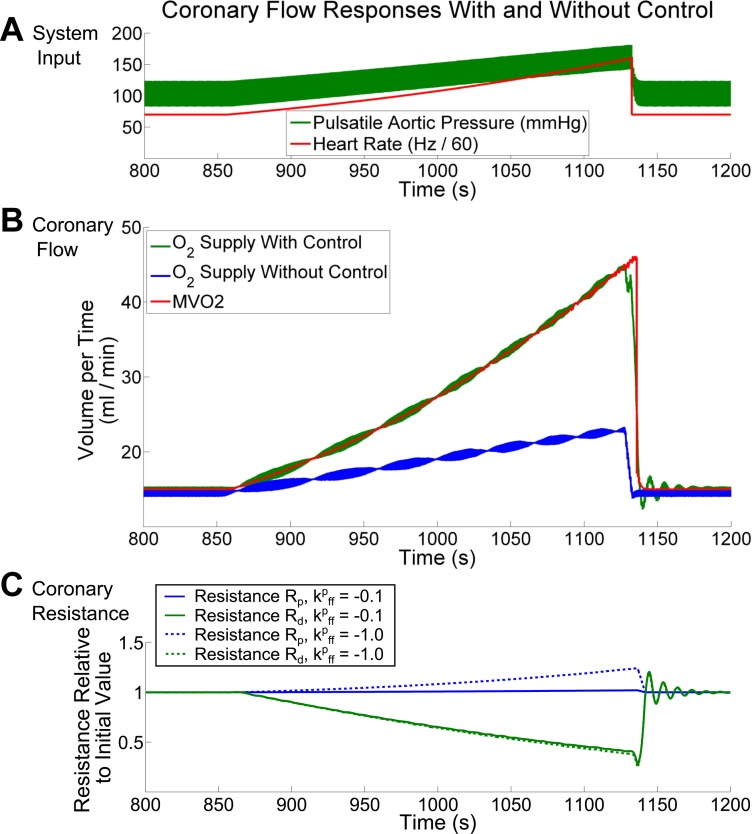
Coronary flow supplying the left ventricle with control on and off. With the imposition of heart-rate and aortic pressure (*A*), the coronary flow increases whether or not control is used (*B*.) However, only with control turned on does coronary flow increase sufficiently to meet the increased demands being placed on the myocardium *C* shows the proportional resistance changes with the α-feedforward gain set to *k*_ff_^p^ = −0.1 (solid lines), and to −1.0 (broken lines); −0.1 was used for *A* and *B*, which are not significantly changed by such an adjustment of *k*_ff_^p^. The overall control primarily affects the microvascular resistance *R*_d_, with a lesser impact on *R*_p_. See *The control response to a ramp of increasing cardiac workload* for further details.

In Fig. 4, we show that control allows for excellent matching between the computed MV̇o_2_ and the myocardial oxygen extraction using synthetically generated input data. By increasing HR and *E*_max_, and thus aortic pressure, over the course of several minutes, we illustrate the controlled response in flow and proportional changes in coronary resistance. For comparison, we also show that without control, despite the modest increase in coronary flow that occurs solely due to the increased myocardial perfusion pressure, the myocardial oxygen extraction remains far from the value of MV̇o_2_ (Fig. 4*B*). This demonstrates the efficacy of our CFCM and its importance for reproducing realistic coronary flows under cardiovascular stress. For the simulation with control turned on, the changes in the controlled resistances are plotted as solid lines in Fig. 4*C*.

When control is turned on, the resistance *R*_d_, affected by both β-feedforward and metabolic feedback control, experiences a resistance drop of ∼60%. In this simulation, the α-feedforward gain, as modeled by *Eq. 7*, is *k*_ff_^p^ = −0.1; this results in the observed 2% increase in *R*_p_ during the simulation. This parameter can be adjusted to investigate the impact of different magnitudes of α-feedforward control on the coronary response to exercise; however, due to the dominance of the metabolic feedback system, the shallow gradients applied to HR and *E*_max_, and because microvascular vasodilator reserve remains available during the entire simulation, using a much larger value of *k*_ff_^p^ = −1.0 does not change the flow results shown in Fig. 4*B*. For comparison, the proportional resistance changes that would occur with *k*_ff_^p^ = −0.1 are shown using broken lines in Fig. 4*C*.

#### Simulating reactive hyperemia.

**Fig. 5.**
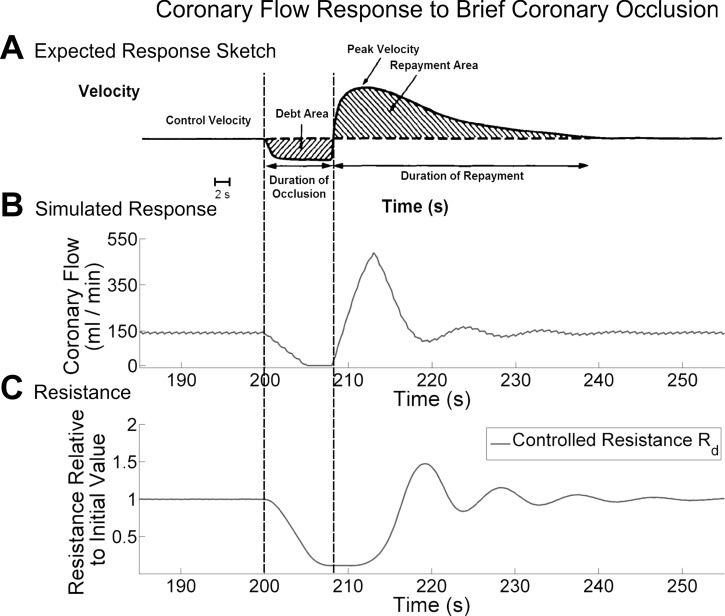
Zero-dimensional simulation. Simulated mean coronary flow in response to an 8-s coronary occlusion (*B*), together with a drawing of the expected response to such an occlusion (*A*), used with permission from Marcus et al. (39). Only the resistance *R*_d_ (*C*) is affected by the control, because only the metabolic control system is activated by the occlusion. Note that the data in *B* is averaged using a 5-s moving window; in reality the initial drop in flow upon occlusion is instantaneous, but the averaging makes it appear to slowly decrease. See *Simulating reactive hyperemia* for further details.

Our CFCM should be able to reproduce aspects of reactive hyperemia; this occurs in response to a historically deficient blood supply, for example after brief coronary occlusion (39). Figure 5 demonstrates this behavior in our model.

We simulate occlusion by holding the coronary flow at 0 for 8 s, before allowing the system to freely respond to the occlusion (Fig. 5*B*). We see that the simulation agrees reasonably well with the expected response also shown in Fig. 5*A*, which is modified from a figure in Ref. 39. However, the CFCM does not reproduce the physiological observation that the total additional volume of blood supplied after the occlusion (Repayment Area in Fig. 5*A*) exceeds the volume of blood that the myocardium was deprived of during the occlusion (Debt Area in Fig. 5*A*); the ratio of these is the repayment ratio and has physiological values in the range 3–4 (39).

Thus Fig. 5 shows that the CFCM reproduces reactive hyperemia to some extent. This will be discussed further in *Reactive hyperemia*.

### Physiological Simulations Using Patient-Specific Cardiac and Coronary Data: Pure Lumped Parameter Model Simulation

We now show that the CFCM is effective using real, patient-specific data. We use data acquired as described in *Human Data for Use in Model Testing*, imposed upon the model as described in that section.

Because the zero-dimensional model provides volumetric flows, while the patient data are in terms of Doppler flow velocities, we compare the two datasets in terms of proportional change relative to their values when the patient is at rest.

**Fig. 6.**
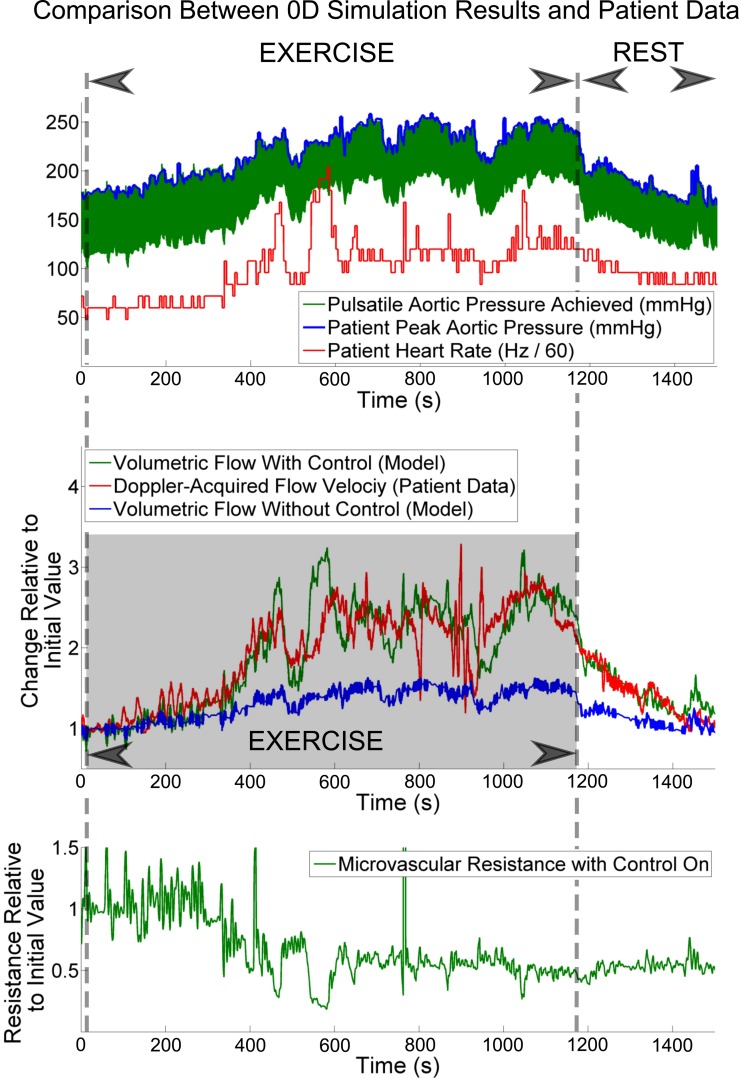
Imposing peak systolic pressure and ECG heart-rate recordings for a patient upon our model (*top*), we observe good agreement between the proportional change in coronary volumetric flow in the model, and the patient-recorded coronary flow velocity (*middle*). Observe also that without control of the coronary Windkessel model, we do not reproduce the patient coronary flow response. This is not surprising, as the microvascular resistance is approximately halved during exercise by the control system when it is active (*bottom*). See *Physiological Simulations Using Patient-Specific Cardiac and Coronary Data: Pure Lumped Parameter Model Simulation* for further details.

The patient was able to exercise for an extended period of time, as can be seen in Fig. 6; it is this, together with the lack of severe coronary stenosis (and thus lack of severely nonlinear coronary resistance), which makes this dataset ideal for testing our CFCM. In the Fig. 6, *top*, we see that the imposition method of *Human Data for Use in Model Testing* performs very well; the peak aortic pressure from the patient agrees closely with the peak aortic pressure achieved by the model. The other imposed variable, the patient's heart rate, is also shown. In Fig. 6, *middle*, we see that the proportional changes in coronary flow predicted by the model agree well with the proportional changes in coronary flow velocity recorded from the patient. This agreement is particularly impressive when we compare it to the blue trace, which shows the flow response in the model, with control turned off. It is likely that the agreement between the model and the patient is even better than this figure shows, because of the difficulty in obtaining in vivo coronary flow velocities; in particular, it is likely that the large-amplitude, short-lived variations in the patient data between 800 and 1,000 s are artifacts of the recording, as opposed to true representations of changes in flow. The controlled change in microvascular resistance is shown in Fig. 6, *bottom*.

### Physiological Simulations using Patient-Specific Cardiac and Coronary Data: Multidomain Simulation

In this section we simulate three-dimensional flow in a model geometry, presenting the results of our CFCM in a multidomain setting (68). Coronary LPN models are coupled to the boundary surfaces of a three-dimensional idealized human aortic and coronary trunk geometry, in which the three-dimensional Navier-Stokes equations are solved. The model has three such coronary boundaries; see Fig. 8 for the geometry labeled *A*, *B*, and *C*. Using the parameterization method described in *Physiological Response to Perfusion Pressure Perturbation: a Parametrization Study*, we determined *g* to be 0.36, 0.46, and 0.47 for the coronary microvasculature downstream to boundaries *A*, *B*, and *C*, respectively. The Young's Modulus of the vessel wall was 3.5 × 103 mmHg (46.6628 N/cm^2^).

The vessel diameters and approximate lengths are provided in Tables 5 and 6.

**Table 5.** Vessel diameters at inflow/outflow surfaces in the model, and at the plane shown in Fig. 8, where flow averaging was performed during evaluation

R	*Coronary A*	*Coronary B*	*Coronary C*	Averaging Plane	Ascending Aorta	Descending Aorta
Diameter, cm	0.19	2.2	0.18	3.6	2.55	1.82

Vessel diameters and approximate lengths are provided in Tables 5 and 6.

**Table 6.** Vessel lengths in the 3-dimensional geometry shown in Fig. 8

	*Coronary A* to Bifurcation	*Coronary B* to Sinus	*Coronary C* to Bifurcation	Aorta
Length, cm	2.5	6.1	5.2	32.2

Distances are for coronary outflow surfaces to their first bifurcation point, unless noted. The length of the aorta is from the aortic valve to the descending aortic outflow.

#### Relative coronary volumetric flow increase under exercise conditions.

**Fig. 7.**
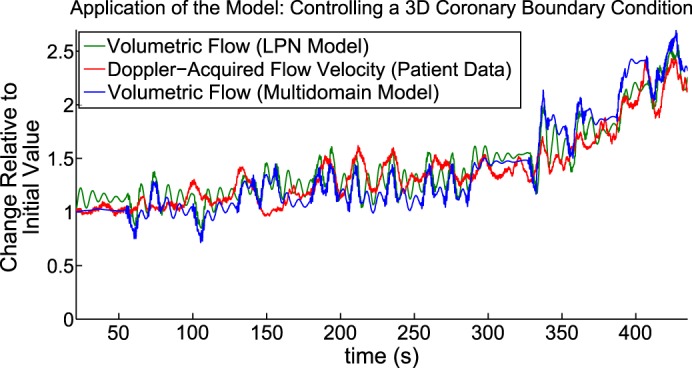
Comparison among LPN simulation, patient data, and multidomain simulation for the first 7 min of Fig. 6. The multidomain flow results agree with the patient data, in terms of proportional changes from the resting state. See *Relative coronary volumetric flow increase under exercise conditions* for further details.

In Fig. 7, we report volumetric blood flow results at *Coronary Boundary A*. This plot uses approximately the first 500 heartbeats of the patient data presented in Fig. 6; this period corresponds to the initial exercise-induced flow increase. As previously, we have reported normalized proportional change in each recording. The patient data are shown in red, the zero- dimensional results are shown in green, and the multidomain results are shown in blue. Note the good agreement between the patient data and simulation results, demonstrating that the CFCM is also applicable in the case of a multidomain simulation. As in the zero-dimensional case, we imposed the peak systolic pressure upon the model, using the method described in *Human Data for Use in Model Testing*. The *E*_max_ imposition surface map for the multidomain simulations is more coarsely sampled than that for our zero-dimensional work; this may explain the small discrepancies that arise between the patient data and multidomain results at higher flows, which can be seen towards the end of the simulation.

#### Absolute coronary flow velocity increase under exercise conditions.

**Fig. 8.**
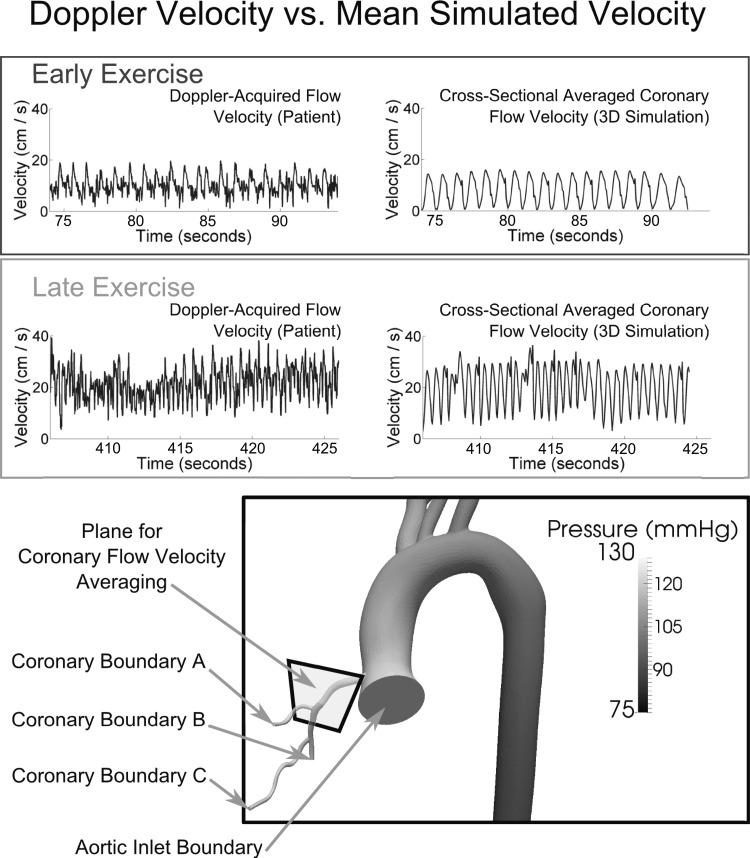
Multidomain simulation. Direct comparison between simulated and patient-recorded coronary flow velocity in the proximal region of the coronary tree, under near-resting flow conditions (early exercise), and at high flow (late exercise) The time scales correspond to those shown in Figs. 7 and 6, and no temporal averaging has been applied to the results The simulated velocity is obtained by taking the velocities in the plane shown at *bottom*, computing their magnitude and averaging over the cross-sectional area. The snapshot of the pressure distribution is at an early point of systole. See *Absolute coronary flow velocity increase under exercise conditions* for further details.

We have so far examined the performance of our CFCM by comparing proportional changes in simulated coronary flow to proportional changes in time-averaged patient flow velocity data. We now demonstrate further validation by examining the simulated local fluid velocity values in the coronary artery, which are available from our multidomain simulations and comparing them with the patient measurements. This direct validation was not possible in the LPN simulations, because flow velocity is not a state variable of such models. We compute the instantaneous magnitude of the velocity in a cross section of the coronary artery, indicated by the plane shown in Fig. 8. We then average this velocity over the area of the cross section and in Fig. 8, *top right*, we report how this velocity changes in time, in both early and late exercise. Compare the results to the patient flow velocity measured with the intracoronary CombowirerXT and reported on the Fig. 8, *top left*; the agreement is good, in terms of amplitude and peak values.

## DISCUSSION

### Summary

We have presented a model for the control of coronary blood flow in response to changing cardiac workload. The model is based on the physiological observations that coronary blood flow closely follows myocardial oxygen demand, that myocardial oxygen debts are repaid, and that control oscillations occur when the system is perturbed. For these reasons, our model is derived from the assumption that the control systems attempt to move the myocardial OSES, *H*(*t*), towards zero. It has a small number of parameters, which we were able to determine from the literature and from existing experimental recordings. It successfully meets its design requirements; in particular, it allows coronary flow to follow the oxygen demand and it reproduces well-established features of coronary flow. It can be used to perform patient-specific simulations, requiring only patient heart rate and aortic pressure as input, and it successfully reproduces recorded coronary flow changes during exercise, both in terms of proportional volumetric flow changes and, in three dimensions, flow velocities. We have shown that it can be applied either as a stand-alone zero-dimensional LPN model or in the setting of a multidomain blood flow simulation.

### Understanding the Control Model

The interpretation of *Eq. 3* is not completely straightforward. It is clear that it allows for damped harmonic motion of *H*, but this motion is complicated by the fact that, in addition, *H* is varying due to *Eq. 2*. To further understand the control system, from *Eq. 3* it is possible to derive the equivalent form

$$\frac{d^{2}Q_{\text{cor}}}{dt^{2}}+g\frac{dQ_{\text{cor}}}{dt}+k_{\text{fb}}\left(t\right)=\gamma^{-1}\left(\frac{d^{2}\text{M}\overset{˙}{\text{V}}{\text{O}}_{2}}{dt^{2}}+k_{\text{fb}}\text{M}\overset{˙}{\text{V}}{\text{O}}_{2}\left(t\right)+g\frac{d\text{M}\overset{˙}{\text{V}}{\text{O}}_{2}}{dt}\right),$$

which shows us that the control is acting to adjust *Q*_cor_ as a damped harmonic oscillator but one that is forced by MV̇o_2_ and its derivatives. If we wish to also see that the control of *Q*_cor_ takes into account historical discrepancies, recorded in the myocardial OSES *H*, we can look at the third equivalent formulation that was given by *Eq. 4*.

### The Damping Coefficient, g, and the Intramyocardial Compliance, C_im_

In *Reproducing coronary flow profiles at rest and under stress*, we explained that the damping coefficient *g* must be tuned manually, dependent on the value of the intramyocardial compliance *C*_im_. We noted that the relationship between these two parameters means that having decreased compliance of the intramyocardial vessels has some equivalence with having increased harmonic damping. This suggests a novel hypothesis for the role of the paradoxical coronary α-feedforward vasoconstriction during exercise, which has been postulated to serve to reduce coronary compliance during exertion. Because α-vasoconstriction operates in vessels exceeding 100 μm in diameter (65), it can be assumed to operate in a portion of the intramyocardial vessels, some of which have a diameter exceeding 200 μm (51), whose compliances contribute to *C*_im_. This suggests that the α-feedforward control system operates to damp feedback control oscillations during perturbations of the cardiac workload and is in agreement with our previous observations using a different CFCM formulation (3). Previous proposed explanations include that α-vasoconstriction acts to reduce wasteful antegrade-retrograde flow oscillation during the cardiac cycle (65), although some workers find no existing explanation to be satisfactory (27).

**Fig. 9.**
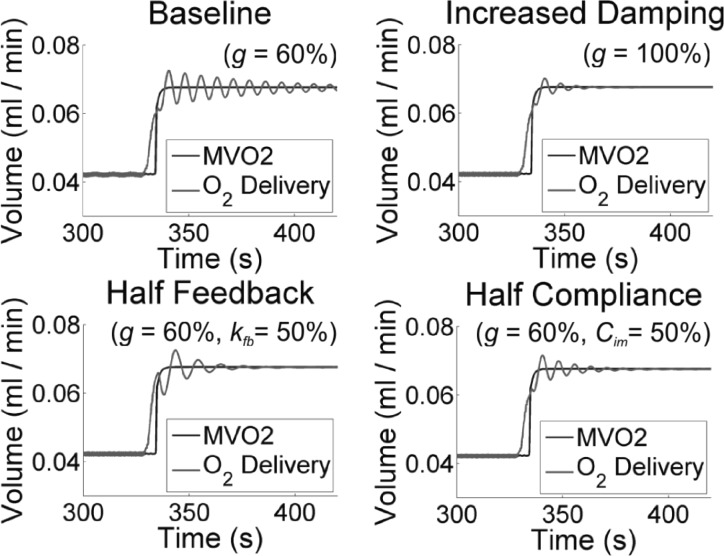
LPN simulation Three strategies for avoiding oscillations caused by the control systems To reveal the oscillations, “Baseline” uses a reduced value of the damping coefficient *g* (=60%), relative to all other zero-dimensional simulations in this work The oscillations can be damped out by either returning *g* to 100% of its original value (“Increased Damping”), or by halving the feedback gain (*g* = 60%; *k*_fb_ = 50%), or by halving the intramyocardial compliance (*g* = 60%; *C*_im_ = 50%). See *The Damping Coefficient, g, and the Intramyocardial Compliance, C*_*im*_ for further details.

Figure 9 demonstrates the relationship between *g* and *C*_im_; under a sharp increase in MV̇o_2_ caused by an enforced instantaneous increase in HR and *E*_max_, the control response is highly oscillatory (“Baseline,” for which *g* is reduced to 60% of the value used in the other zero-dimensional simulations in this article, to reveal the oscillatory phenomenon). The remaining three panels of Fig. 9 show three simulations with different parameters changed to damp the oscillatory behavior; respectively, returning the damping coefficient to 100% (“Increased Damping”), reducing the feedback gain (“Half Feedback”), and halving *C*_im_ (“Half Compliance”). Note that reducing the feedback gain *k*_fb_ is not an ideal option, as it leads to a longer initial period during which O_2_ extraction is less than MV̇o_2_.

There are two points to understand regarding this. The first is that, due to their interactions, parameterization of *g* must be performed after selecting the appropriate value of *C*_im_. The second is that one role of α-feedforward vasoconstriction at the onset of exercise may serve to help damp such oscillations via a compliance-reduction effect.

We have not attempted to include automatic control of *C*_im_ in our CFCM; a simple approach might be to adjust *C*_im_ in inverse proportion to MV̇o_2_, which we take here as a proxy for sympathetic α-activation. Studies of noncoronary arteries indicate that the α-adrenoceptor induced compliance reduction can be 30–40% (26, 60).

### Model Sensitivity to Parameter Variation

By designing the CFCM to have a small number of parameters, we have followed the principle of parsimony and therefore do not believe that it suffers from overfitting (40). A related consideration is that simulation results using the CFCM should not be highly sensitive to changes in the exact model parameters used. When comparing to patient data, the sensitivity of our CFCM to parameter variation is low; modifying either g or *k*_fb_ by a factor of two in either direction does not cause significant changes in the agreement between coronary flow in the model and in patient recordings. However, increasing the damping coefficient *g* does reduce the speed at which the system responds to perturbations and decreasing it would make experiments such as those shown in Fig. 2 less stable. Similarly, increasing the feedback gain *k*_fb_ would cause the flow in Fig. 2 to be excessively oscillatory, and decreasing it reduces the ability of the system 1 to respond to rapid changes in MV̇o_2_. These changes correspond exactly to the expected physiological meaning of the parameters, and we conclude that the sensitivity of our model is appropriate.

### Feedforward Control

#### Adrenergic feedforward.

An interesting feature of *Eq. 6* is that one of its terms, $\gamma^{-1}\frac{d\text{M}\overset{˙}{\text{V}}{\text{O}}_{2}\left(t\right)}{dt},$ is not strictly part of a feedback system, because it is not an error signal; rather, it is a perturbation that could cause the OSES to increase and can therefore be interpreted as a term corresponding to the β-feedforward control. In previous work, where a simple coronary flow control model was investigated, this term was introduced to include feedforward control in the model (3). In the present work, by beginning with a harmonic model, the β-feedforward term has arisen naturally, and so it could be argued that under our assumptions, we have derived the requirement for feedforward control. Thus, before any reference to simulation, our CFCM suggests the following hypothetical viewpoint: neural feedforward control of the coronary arteries exists to make the control of coronary blood flow approximately harmonic in nature. However, it must be noted that this requires the additional modeling assumption that the level of feedforward coronary control signaling is directly related to $\frac{d\text{M}\overset{˙}{\text{V}}{\text{O}}_{2}\left(t\right)}{dt}$, which is only likely to be valid under circumstances where the sympathetic nervous system alone is responsible for changes in MV̇o_2_. However, because our CFCM does not explicitly include the autonomic nervous system, we are unable to account for this, but it is possible to imagine that the different systems are tuned to achieve this harmonic-type control.

**Fig. 10.**
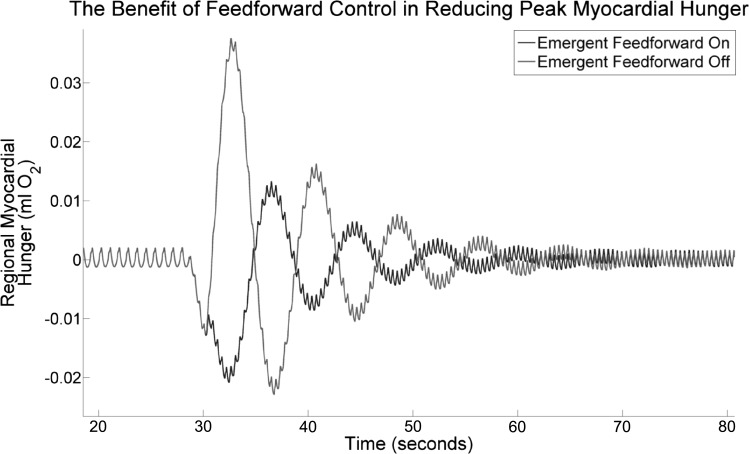
LPN simulation. The myocardial oxygen supply error signal (OSES) after a strong perturbation in HR and *E*_max_, with and without the emergent β-feedforward control system switched on. Without the feedforward system, the myocardial OSES peaks at a value around three times higher than when feedforward is present. Note that the 2 traces agree until around 30 s. See *Adrenergic feedforward* for further details.

A more direct point of view, and one noted previously in the literature, is that feedforward control improves the speed and accuracy of the coronary flow response to exercise. Our results agree with this, as we show in Fig. 10. Starting with a system in equilibrium, a sharp change in HR and systolic pressure is enforced, doubling the HR and increasing the peak systolic pressure by ∼40%. Figure 10 shows how the myocardial OSES evolves, while the control systems adapt the coronary resistance to this change. Two simulations were performed, one with β-feedforward present, and the other with it turned off; this was achieved by removing the term in MV̇o_2_(*t*) from *Eq. 6*. We see that without β-feedforward, the myocardial OSES peaks at a value around three times larger than it would have done otherwise and the system takes marginally longer to adjust to the new equilibrium. A more precise comparison is given by the integral of the absolute value of OSES histories shown in Fig. 10; this “discrepancy” integral is 1.86 × 10^−1^ ml O_2_/s with β-feedforward on, and 2.92 × 10^−1^ ml O_2_/s with β-feedforward off, demonstrating that in this experiment β-feedforward control resulted in a 40% reduction in this discrepancy. We note that this absolute value integral treats O_2_ oversupply and undersupply as being equally undesirable; this is reasonable, because the control systems actively attempt to avoid both under- and overperfusion. A possible explanation for this avoidance is that overperfusion can induce an increase in MV̇o_2_, and a reduction in myocardial mechanical efficiency, via the Gregg effect (56). As further evidence, we note that the Gregg effect is considered to be unimportant in the left ventricle under physiological conditions, precisely because the coronary control systems prevent the perfusion changes that would cause it (71).

#### A metabolic feedforward control hypothesis.

An alternative hypothesis for the metabolic control mechanism was put forward by Saitoh et al. (53). This states that cardiac myocytes produce H_2_O_2_ at a rate proportional to their O_2_ consumption and that it is this H_2_O_2_ that causes vasodilation. A simple mathematical expression of this is given as follows. If we let [H_2_O_2_](*t*) be the concentration of H_2_O_2_ at time *t*, we can express how this changes as the difference between a production term (in proportion to MV̇o_2_) and a breakdown term (as an exponential decay-type term, in keeping with the description given by Saitoh et al.),

(9) $$\frac{d\left[{\text{H}}_{2}{\text{O}}_{2}\right]}{dt}=k_{1}\text{M}\overset{˙}{\text{V}}{\text{O}}_{2}\left(t\right)-k_{2}\left[{\text{H}}_{2}{\text{O}}_{2}\right]\left(t\right).$$

for some unknown positive constants *k*_1_ and *k*_2_. Under this control hypothesis, if flow *Q*(*t*) is to be proportional to [H_2_O_2_](*t*), for some positive proportionality constant α we can write

(10) $$Q\left(t\right)=\alpha \left[H_{2}{\text{O}}_{2}\right]\left(t\right).$$

Upon combining these two equations, we obtain

(11) $$\frac{dQ}{dt}=\alpha k_{1}\text{M}\overset{˙}{\text{V}}{\text{O}}_{2}\left(t\right)-k_{2}Q\left(t\right).$$

This is essentially already one of the contributing terms in our control equation (the $\frac{dH\left(t\right)}{dt}$ term in *Eq. 4*) that we obtained during our original derivation. Thus, such behavior exists in our model, and although we do not have data to determine the parameters, we would expect that they would be similar to the effective values of these terms within our control equation, simply because our control model reproduces physiological behavior.

### The Benefits of Three-Dimensional Simulations

In *Physiological Simulations using Patient-Specific Cardiac and Coronary Data: Multidomain Simulation*, we performed what is likely the longest simulation of aortic-scale, three-dimensional, patient-specific hemodynamics reported in the literature to date. Five hundred heart-beats were reproduced, covering some 422 s of real-time. This simulation took approximately 2 wk using 256 cores of an SGI UV 1000 high-performance computing system.

The benefit of this approach, and the justification for deploying such computational power, were that it enabled us to make direct comparisons between the flow velocities recorded in the patient and the flow velocities produced by the simulation, and a good agreement between these was demonstrated, both early and late in the exercise period covered. Indirectly, this provides evidence that it was reasonable to compare proportional changes in coronary flow velocity and volumetric flow in the LPN simulations; that is, the proportional comparisons between patient flow velocity and simulated mass delivery shown in, for example, Fig. 7 displayed good agreement, and we see in Fig. 8 that this good agreement is indeed borne out in terms of the actual velocity in the coronaries.

Additionally, these simulations confirmed the applicability of our CFCM when performing multidomain coronary simulations, which are of interest in the assessment of the clinical significance of coronary stenoses (41). Three-dimensional simulation is mandatory whenever complicated vessel geometry has a large impact on the hemodynamics. A prime example is the simulation of severe coronary stenosis, where we must capture the nonlinear viscous losses across the stenosis.

### Comparison with Previous Efforts

#### Differences in model design.

An important previous model relating coronary flow to MV̇o_2_ is that of Dankelman et al. (12), which builds on the algebraic model of Drake-Holland et al. (19). The core of the Dankelman et al. model can be expressed as a second-order ordinary differential equation for the changing resistance; doing so reveals that, coronary resistance varies when there is an instantaneous mismatch between oxygen delivery and consumption. This assessment of mismatch is instantaneous only, which means that it cannot account for any previous supply inadequacies. Our control equation is an ordinary differential equation for the changing coronary flow, it is derived from a different premise (that the OSES undergoes a damped harmonic motion), and it accounts for historical supply inadequacies. The historical-tracking aspect of our model is always active, and the clearest demonstration of its importance comes from the fact that our model can reproduce reactive hyperemia. Additionally, the derivation of our model naturally revealed feedforward-like terms in the control equation, which has not been observed in previous models.

#### Dynamic control responses.

In addition to the dynamic responses studied in the present work, previous work on modeling coronary responses to perturbations of pressure, flow or MV̇o_2_ includes the normalized resistance model of Dankelman et al. (12) In light of these studies, We performed additional investigations with our model, finding that it reproduces the observation that the response time of coronary resistance to 20% changes in MV̇o_2_ is slower with constant flow perfusion than with constant pressure perfusion (12). With a single set of parameters, our model can reproduce differing speeds of response to constant-MV̇o_2_ with pressure steps up or pressure steps down as observed in goats (13). This is an aspect that the model of Dankelman et al. could not reproduce without using different model parameters when stepping up than when stepping down. The present model can do this with the same model parameters in both directions, although the parameters must be adjusted to achieve this; this is not surprising as we originally parameterized using dogs rather than goats, and it has been noted that the phenomenon in question is absent in dogs (15). Our model did not reproduce their reported differences between normalized resistance responses at different constant perfusion pressures (12).

### A Potential Application: Detecting Anomalous Coronary Control Responses

It may be possible to use the CFCM to identify pathological coronary perfusion by comparing patient-specific simulation and patient-recorded coronary flow. Since the CFCM always seeks to eliminate myocardial OSES, we would not expect it to make accurate predictions of coronary flow for a patient whose myocardium became ischemic during exercise, unless we were able to discover the patient-specific minimum coronary microvascular resistance and impose it upon the model. In the absence of microvascular resistance data, however, we can hypothesize that the point of divergence between the model predictions and the patient data could indicate the point of onset of ischemia.

Conversely, the fact that our CFCM was able to accurately predict changes in coronary flow in the case studied in the present work suggests that O_2_ extraction by the myocardium remained largely sufficient throughout the diagnostic procedure. This is supported by the fact that the patient was able to exercise for ∼20 min.

### Model Limitations

#### Reactive hyperemia.

As noted in *Simulation reactive hyperemia*, while the CFCM does reproduce reactive hyperemia in part, it only pays back the exact coronary flow debt that has been incurred, without the “interest,” which would manifest as the repayment-to-debt ratio seen in Fig. 5*B*. While the repayment in terms additional hyperemic blood flow to flow debt should exhibit this additional repayment, there is evidence to suggest that the oxygen repayment ratio may be closer to unity (52). The CFCM evaluates oxygen delivery and we keep it constant, so this lack of repayment interest in flow reflects the lack of repayment interest in oxygen, which is more physiological. This observation suggests that it may be beneficial to introduce a tissue oxygen model to adjust dynamically or an integration kernel to *Eq. 2* that penalized more long-standing oxygen debt differently. However, the importance of doing this would depend on the particular application for the CFCM.

#### Extreme arterial pressure.

The CFCM attempts to provide sufficient coronary flow for a given MV̇o_2_ and will succeed in this if sufficient vasodilatory reserve is available, regardless of the perfusion pressure. Physiologically however, for a given MV̇o_2_ the flow will vary a small amount with changing perfusion pressure, with larger variations at very high or very low pressure (66). This phenomenon could be integrated into the CFCM by using a family of sigmoidal pressure-autoregulation curves of coronary flow against perfusion pressure, with each particular curve associated with a given MV̇o_2_ range; there is experimental evidence supporting such an approach (44).

#### Ischemia.

Physiologically, if MV̇o_2_ is computed as coronary flow multiplied by the difference between arterial and venous O_2_ content, then the obtained value for MV̇o_2_ is only valid in the absence of ischemia (63). In the present work, we do not compute MV̇o_2_ in this way; we derive MV̇o_2_ from the PV loop of the simulated heart model, and it so should be a good approximation of the myocardial oxygen demand. In the case where it is impossible for MV̇o_2_ to be satisfied by coronary flow [i.e., the simulated coronary vasculature has reached maximal (patho-)physiological vasodilation, but our PV-loop-computed MV̇o_2_ remains unsatisfied by the flow], the OSES will increase unboundedly, but cardiac performance will not be affected, as this is not something which our model is designed to reproduce.

### Potential for Further Model Development

#### Cardiac efficiency.

In *Computing the Myocardial Oxygen Consumption*, we explained the method for relating the left-ventricular PV loop to myocardial oxygen consumption. In particular, we took the ratio of PVA to MV̇o_2_ to be one-third. This was taken from an existing study (29) and is an average computed from 11 patients; if we look at the data itself, we see that it decreases approximately linearly with increasing cardiac inotropic state (the slope of the end-systolic PV relationship) and that it ranged between 0.2 and 0.4 (29). Thus it may be important to improve our CFCM by making PVA(*t*)/MV̇o_2_(*t*) a function of inotropy.

#### Coronary-extractible blood oxygen content.

In this work, we have assumed constant arterial and coronary venous oxygen contents, so that we have a fixed value of the coronary extractible blood oxygen content, γ. Venous blood oxygen content does not vary much; one dataset shows dog venous oxygen partial pressure to be 20 mmHg (0.2666 N/cm^2^) at rest, and 16 mmHg (0.2133 N/cm^2^) with paired pacing, but this 4-mmHg (0.0533 N/cm^2^) difference should be seen in the context of the arterial oxygen partial pressure, which was 116 mmHg (1.5465 N/cm^2^) in both cases (42). Including a model for this variation would be possible, but it is not clear that it would add much value to the CFCM. Indeed, we experimented with changing to be 0.2 (large extraction) and 0.05 (small extraction). We observe that with increasing, the oscillatory nature of coronary resistance, and thus of the coronary flow, increases in magnitude slightly, but the feedback mechanism always ensures that MV̇o_2_ is satisfied (so long as the vasodilatory reserve is not exhausted). Naturally, therefore, if we double the extraction, the mean volumetric coronary flow halves in the model. Incidentally, this highlights that our original choice of γ = 0.125 was a good one, as in our three-dimensional simulation it allowed us to correctly reproduce the flow velocities observed in the patient. An incorrect value for would have caused these simulated velocities to be incorrect.

#### Separation of multiple control systems.

It may be important to attempt separation of the myogenic, shear, and metabolic control systems. The reason for doing this is that currently the CFCM increases the coronary resistance when the myocardial OSES is negative; this occurs when myocardial perfusion exceeds metabolic requirements. In this article, we have capped this negative myocardial OSES at a value corresponding to ∼0.5 s of resting cardiac O_2_ requirements. Reducing the magnitude of this cap reduces the oscillatory behavior of the control system, meaning that the reactive hyperemia response shown in Fig. 5 is less oscillatory and thus more physiological. However, this also implies that the agreement in oscillatory amplitude between the CFCM and the pressure drop experiment shown Fig. 2 would be diminished and therefore less physiological. Separation of control systems may allow us to explain this discrepancy but at the cost of increasing the number of CFCM parameters that must be determined.

### Conclusion

We have presented a new coronary flow control model that allows us to reproduce the changes seen in patient-recorded coronary blood flow during exercise. The control is a function of changes in myocardial oxygen demand, computed automatically from a lumped parameter model of the heart. It is based on harmonic motion of the myocardial OSES, and acts by attempting to drive this OSES towards zero. It has sufficient parameters to reproduce interesting physiological phenomena, but sufficiently few so that that parameterization is straightforward. It is capable of producing a number of physiologically observed phenomena, and can either be used as part of a single lumped-parameter model of the heart, coronary arteries and systemic vessels (Fig. 1), or to create a boundary condition at the coronary boundaries of the three-dimensional domain in a multidomain model. From the input of only the heart rate and peak aortic pressure for a patient, it is possible to reproduce the proportional changes in coronary flow observed in that patient; this is not possible without a control model, as was seen in Fig. 6. The ability to predict the expected changes in flow during exercise raises the possibility of comparing patient recordings with model predictions to detect flow insufficiencies, such as at the onset of ischemia, or measurement anomalies such as changes of the insonation angle of the coronary artery by the Doppler wire during acquisition. The derivation of the model has shown that β-feedforward adrenergic control is mathematically necessary for harmonic control of the coronary flow and has generated a novel hypothesis for the resolution of the α-vasoconstriction paradox. In general, the model should be used in hemodynamic simulations that include a heart model and coronary vasculature as a boundary condition, both to improve physiological accuracy and to automatically and dynamically determine the appropriate coronary resistance without the need for significant user intervention.

## GRANTS

We gratefully acknowledge support from the European Research Council under the European Union's Seventh Framework Programme (FP/2007–2013)/ERC Grant Agreement 307532 and the United Kingdom Department of Health via the National Institute for Health Research (NIHR) comprehensive Biomedical Research Centre award to Guy's & St Thomas' NHS Foundation Trust in partnership with King's College London and King's College Hospital NHS Foundation Trust. K. Asrress is supported by a British Heart Foundation Clinical Training Fellowship (FS/11/43/28760).

## DISCLOSURES

Conflict of interest statement: No conflicts of interest, financial or otherwise, are declared by the author(s).

## AUTHOR CONTRIBUTIONS

Author contributions: C.J.A., K.D.L., K.N.A., S.R.R., and C.A.F. conception and design of research; C.J.A., K.N.A., and S.R.R. performed experiments; C.J.A. analyzed data; C.J.A. and C.A.F. interpreted results of experiments; C.J.A. prepared figures; C.J.A. drafted manuscript; C.J.A., K.D.L., K.N.A., and C.A.F. edited and revised manuscript; C.J.A. and C.A.F. approved final version of manuscript.

## Footnotes

1 Autoregulation is defined to be the ability to maintain constant blood flow, regardless of perfusion pressure changes, and in the absence of changes in tissue oxygen demand (20).
